# Wafer-Scale LSPR Substrate: Oblique Deposition of Gold on a Patterned Sapphire Substrate

**DOI:** 10.3390/bios12030158

**Published:** 2022-03-03

**Authors:** Kihyeun Kim, Ki Joong Lee, Na Rae Jo, Eun-Jung Jo, Yong-Beom Shin, Min-Gon Kim

**Affiliations:** 1 Center for Systems Biology, Massachusetts General Hospital, Boston, MA 02114, USA; kkim50@mgh.harvard.edu; 2 Bionanotechnology Research Center, Korea Research Institute of Bioscience and Biotechnology (KRIBB), 125 Gwahak-ro, Yuseong-gu, Daejeon 34141, Korea; kijoong123@kribb.re.kr (K.J.L.); nalgamgi@kimm.re.kr (N.R.J.); 3 Department of Chemistry, Gwangju Institute of Science and Technology (GIST), Gwangju 61005, Korea; jej@gist.ac.kr; 4 BioNano Health Guard Research Center, 125 Gwahak-ro, Yuseong-gu, Daejeon 34141, Korea

**Keywords:** patterned sapphire substrate, localized surface plasmon resonance, oblique deposition, nanoimprint lithography, wafer-scale

## Abstract

Label-free detection of biomolecules using localized surface plasmon resonance (LSPR) substrates is a highly attractive method for point-of-care (POC) testing. One of the remaining challenges to developing LSPR-based POC devices is to fabricate the LSPR substrates with large-scale, reproducible, and high-throughput. Herein, a fabrication strategy for wafer-scale LSPR substrates is demonstrated using reproducible, high-throughput techniques, such as nanoimprint lithography, wet-etching, and thin film deposition. A transparent sapphire wafer, on which SiO_2_-nanodot hard masks were formed via nanoimprint lithography, was anisotropically etched by a mixed solution of H_2_SO_4_ and H_3_PO_4_, resulting in a patterned sapphire substrate (PSS). An LSPR substrate was finally fabricated by oblique deposition of Au onto the PSS, which was then applied to label-free detection of the binding events of biomolecules. To the best of our knowledge, this paper is the first report on the application of the PSS used as an LSPR template by obliquely depositing a metal.

## 1. Introduction

Localized surface plasmon resonance (LSPR), which occurs in metal nanostructures, has attracted considerable attention from various optical- and electrical-based research fields, such as optical biosensors, color engineering, spectroscopy, metamaterials, and plasmonic optoelectronics [1,2,3,4,5]. Thus, large-scale, reproducible, high-throughput fabrication methods of wavelength-tunable LSPR substrates are in high demand not only for research but also for commercialization [6,7,8]. To fabricate LSPR substrates, two primary methods have been used until now: (i) the synthesis of metal nanoparticles (NPs), and covalent attachment of the NPs on substrates through self-assembled monolayers (SAMs) [9,10]; and (ii) the patterning of metal nanostructures by nanolithography techniques [7,11,12].

Regarding the former (i), various shapes of gold (Au) NPs were synthesized in a solution, such as spheres, rods, triangles, and spiked structures, which could easily tune LSPR properties depending on the shape [13,14]. However, the formation of SAMs on a substrate was usually unstable, which lowered reproducibility for the fabrication of LSPR substrates [15,16]. Moreover, it was difficult to align metal NPs on a substrate [17]; instability issues when metal NPs were immobilized on a substrate were also noted [18,19]. As for the latter (ii), various nanolithography techniques, such as colloidal, e-beam, and nanoimprint have been applied to fabricate metal nanostructures on substrates [20,21,22]. The limitations of e-beam and colloidal lithography are low-throughput and non-elaborate patterning, respectively [23]. Although nanoimprint lithography enabled a high-throughput process and elaborate patterning, it also encountered difficulties in the fabrication of various shapes of master molds, leading to limitations in the tuning of LSPR properties [24]. 

Herein, we report on a fabrication method for wafer-scale, elaborate LSPR substrates using high-throughput methods, such as nanoimprint lithography, wet-etch processing, and thin film deposition. In detail, the LSPR substrates were accomplished by oblique deposition of Au onto a (triangular-pyramid shape) patterned sapphire substrate (PSS) which was obtained through a wet-etch process, resulting in the formation of Au nanostructures on one side of the PSS. Thus, unlike typical strategies for fabrication of LSPR substrates that directly fabricated patterned metal nanostructure using nanolithography [20,21,22], the strategy suggested in this paper for LSPR-substrate fabrication was to pattern a substrate first, followed by oblique deposition of Au on the patterned substrate. This approach can variously tune LSPR properties according to the shape of PSS controlled by the wet-etch condition, deposition angle and/or direction of metals, which would be highly advantageous for obtaining wavelength-tunable LSPR substrates. Therefore, such a fabrication method could be an inspiring approach for nanofabrication and/or LSPR-based research fields.

## 2. Materials and Methods

### 2.1. Formation of a SiO_2_-Nanodot on the Sapphire Wafer

SiO_2_-nanodot was formed on a sapphire wafer (2 inches in diameter) via nanoimprint lithography, as reported in our previous study (Figure S1) [23]. In brief, a nanodot-patterned master stamp was used to pattern a thermoplastic resist layer that had been coated on the sapphire wafer. Then, Cr was obliquely deposited on the resist pattern to form the Cr hard masks only on the top of the resist layer that was not stamped by the master stamp. Next, O_2_ plasma was treated to etch the resist layer where it was not covered by the Cr hard mask. A SiO_2_-nanodot pattern was formed by the deposition of SiO_2_ using an e-beam evaporator. Finally, the resist layer was completely removed using acetone. Such nanoimprint lithography is a highly advantageous technique for fabricating nanosized patterns on substrates because of its high speed and high-throughput, as well as high reproducibility and fidelity [23]. The diameter, height, and pitch of the SiO_2_ nanodots were 130, 50, and 300 nm, respectively.

### 2.2. Fabrication of PSS via Wet-Etch

The SiO_2_ nanodot-deposited sapphire wafer was annealed using a furnace under air at 750 °C for 12 h to densify the SiO_2_. Then, the sapphire wafer was soaked in a beaker containing a mixture of H_2_SO_4_ and H_3_PO_4_ (1:3 *v*/*v*), and heated for 1 h at 310 °C to etch the sapphire wafers [25,26]. After finishing the wet-etch process, the beaker was cooled to 20 °C, and the PSS was washed with deionized (DI) water.

### 2.3. Formation of Metal Nanostructures on the PSS 

Au nanostructures were formed on the PSS by oblique deposition (45°) of Au using a thermal evaporator. The deposition thickness and rate were set to 20 nm and 0.5 Å/s, respectively.

### 2.4. LSPR Shift of the Au Nanostructure-Formed PSS Due to Biomolecule Attachment

An LSPR shift was observed by exposing it to streptavidin (STA), biotin-bovine serum albumin (BSA), and STA, in turn. To attach STA onto the Au surface, 100-µM biotin-HPDP was attached to the Au nanostructure on the PSS overnight, followed by washing of the Au/PSS with EtOH and deionized (DI) water. Then, STA (50 µg mL^−1^) and biotin-BSA (50 µg mL^–1^) were attached in turns; and at each step, absorbance was measured after washing.

## 3. Results and Discussion

**Figure 1:**
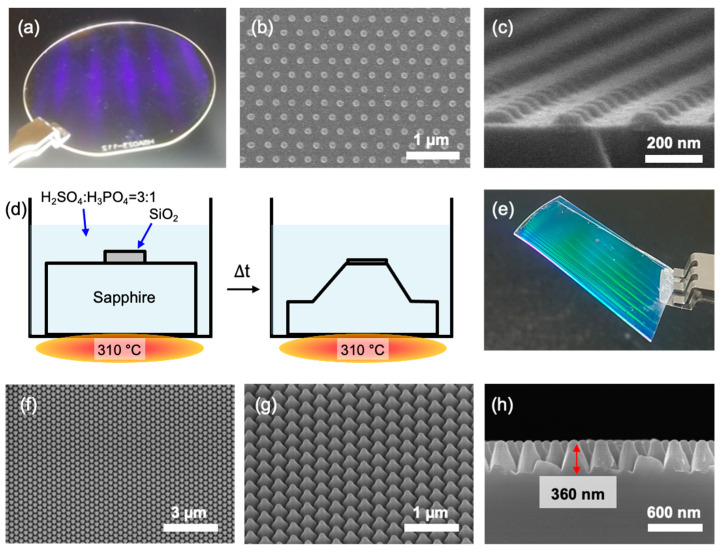
(**a**) A digital image and (**b**,**c**) SEM images of the SiO_2_ nanodot array on the sapphire wafer (2 inches) ((**b**) top and (**c**) tiled view). (**d**) Schematic of the fabrication of the patterned sapphire substrate (PSS) via wet-etch. (**e**) A digital image of a piece of the PSS. (**f**–**h**) SEM images of the PSS for top, 45° tilted, and side views, respectively.

To fabricate a patterned sapphire substrate (PSS), SiO_2_ nanodots—which played a role as hard masks—were well-orderly formed on the entire surface of the sapphire wafer (Figure 1a–c). Figure 1a shows blue light scattering observed when the sapphire wafer was exposed to light due to the well-ordered SiO_2_ nanodots on the wafer. In addition, SEM images proved that precisely ordered SiO_2_ patterns were present on the sapphire wafer; the diameter, height, and pitch of the pattern were 130, 50, and 300 nm, respectively (Figure 1b,c).

The SiO_2_ nanodot pattern on the sapphire wafer was annealed using a furnace under air at 750 °C for 12 h to densify the SiO_2_ nanodots before proceeding with the wet-etch process. This process was essential because as-deposited SiO_2_ nanodots using the e-beam evaporator were not able to act as hard masks under high temperatures and acidic conditions (such as a mixture of H_2_SO_4_ and H_3_PO_4_), as shown in Figure S2. After the annealing process, the sapphire wafer was wet etched by a mixed solution composed of H_2_SO_4_ and H_3_PO_4_ at an elevated temperature of 310 °C for 1 h (Figure 1d). The composition of a wet-etching solution was 3:1 *v*/*v*.

As a result of the wet-etch, a PSS was fabricated, which was analyzed by digital (Figure 1e) and SEM images regarding top, 45° tilted, and side views (Figure 1f–h). Triangular-pyramid shape patterns in the PSS were observed across the entire wafer (Figure 1f,g). The triangular pyramid could have been formed because the SiO_2_ hard mask prevented direct contact between the single crystal sapphire wafer and acidic etchant (H_2_SO_4_ and H_3_PO_4_). The height of the triangular pyramids on the PSS was approximately 360 nm (Figure 1h), which varied depending on the wet-etch temperature (Figure S3). This process occurred because although H_2_SO_4_ and H_3_PO_4_ can etch sapphire as follows (Equations (1) and (2)):

Al_2_O_3_ + 3H_2_SO_4_ → Al_2_(SO_4_)_3_ + 3H_2_O, (1)

Al_2_O_3_ + 2H_3_PO_4_ → 2AlPO_4_ + 3H_2_O, (2)

the roles of both etchants are different in terms of etching direction (H_2_SO_4_ and H_3_PO_4_ for perpendicular and lateral, respectively) [25]. In addition, these different behaviors also depended on the temperature. Thus, shape control was achieved by controlling the composition of the etchant. In addition, after the first etch of the sapphire wafer, the second etch of the wafer using a different composition of etchant resulted in sharp or broad triangular-pyramid shapes on the PSS (Figure S4).

**Figure 2:**
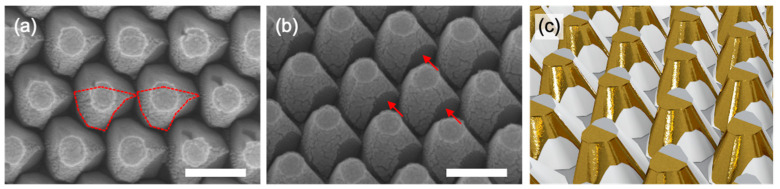
(**a**,**b**) SEM images after oblique (45°) deposition of Au onto a PSS ((**a**): top view, (**b**): 45° tilted view). Scale bars in the images are 300 nm. Red lines in (**a**) and arrows in (**b**) indicate Au nanostructure and the shadowing effect due to adjacent triangular pyramids, respectively. (**c**) Schematic illustration of obliquely deposited (45°) Au on PSS.

**Figure 3:**
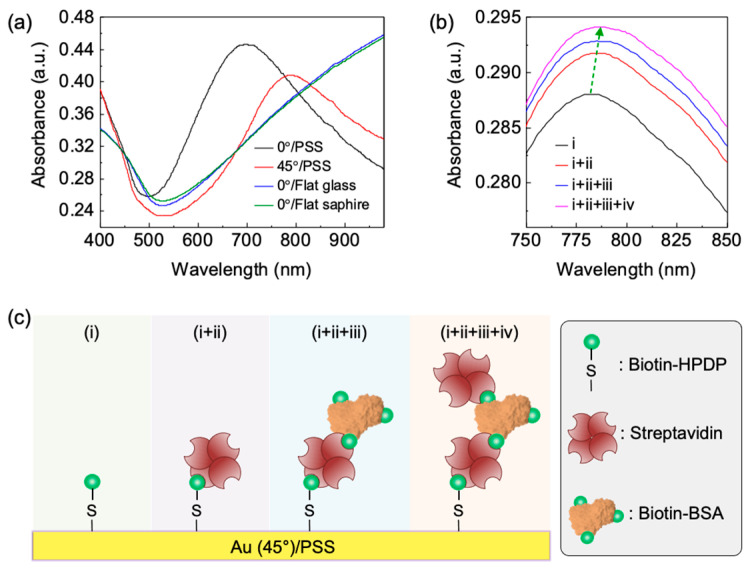
(**a**) Absorbance of obliquely and vertically deposited Au on PSSs, and vertically deposited Au on flat glass and sapphire substrates. (**b**) LSPR shift of the PSS with obliquely deposited Au: (i) biotin-HPDP, (ii) STA, (iii) biotin-BSA, and (iv) STA. (**c**) Schematic illustration for step-by-step biomolecule detection using an Au/PSS. The stages of (i), (i + ii), (i + ii + iii), and (i + ii + iii + iv) correspond to those in Figure 3b.

Wafer-scale Au nanostructures were fabricated by oblique deposition of Au (20 nm) on the PSS using an evaporator. Oblique deposition (45°) of Au on the PSS resulted in Au nanostructures being only formed on only half of the available surface area of the triangular pyramids on the PSS, described as red lines in Figure 2a; moreover, a shadowing effect was observed due to adjacent triangular pyramids (red arrow in Figure 2b). For better visualization, a schematic illustration was demonstrated for obliquely deposited Au (45°)/PSS (Figure 3c). Whereas, vertical deposition resulted in nearly full coverage of Au on the PSS (Figure S5). In the case of oblique deposition of Au, the shape of the Au nanostructures was determined by the shape of triangular pyramids on the PSS, which can be controlled by the composition of etchant and etching temperature (Figures S3 and S4). Consequently, the shape of Au nanostructure can vary not only depending on the shape of the triangular pyramids on the PSS but also the deposition angle and/or direction with respect to the PSS.

The phenomenon of a localized surface plasmon resonance (LSPR) of the Au nanostructures on the PSS was observed by measuring absorbance (Figure 3a). LSPR peaks were observed in the cases of Au deposition on a PSS, but not on flat substrates. The LSPR peak appeared in the vertically deposited case because the vertical deposition between the sample and Au sources in the thermal evaporator used in this work was not exactly vertical (Figure S6). Regarding the obliquely deposited case, the LSPR peak was red-shifted compared to that from the vertically deposited case; this was because the shapes of Au nanostructures in each case were different. Different shapes of metal nanostructure induce various LSPR properties [27]. Therefore, the location of the LSPR peak can be controlled by the shape of triangular pyramids on the PSS and deposition angle and/or direction of the PSS.

One of the most popular applications of the LSPR phenomenon is in biosensing fields because the LSPR shift occurs by perturbation in the presence of target molecules located near the metal nanostructures (~10 nm), which is a label-free tool that monitors molecular bindings in real-time, in small-volume samples [28]. Hence, Au nanostructures on PSS were exposed to biomolecules (STA, biotin-BSA, and STA, in turn), and LSPR shifts were observed (Figure 3b). A schematic illustration is depicted for the procedure for biomolecule detection (Figure 3c). In detail, to attach STA on the surface of the Au nanostructures, biotin-HPDP was, at first, covalently functionalized on the Au surface through the thiol-Au interaction [29]. As a result of the step-by-step attachment of STA/biotin-BSA/STA, the LSPR peak of the Au nanostructures was red-shifted step-by-step due to the attachment of the biomolecules (detailed values of LSPR are shown in Table S1). The redshift phenomenon occurred because the biomolecules attached to the Au nanostructures caused a change in the refractive index near the Au surface. Thus, our Au nanostructure on PSS could have an application as LSPR biosensors by manipulating the structures of the PSS and/or oblique deposition conditions. Furthermore, a thorough investigation of LSPR properties by controlling the PSS and deposition variables, such as the plasmonic substrate, could be further applicable to color engineering, spectroscopy, metamaterials, and plasmonic solar cells.

We also fabricated Au/PSSs where Au was deposited at different angles: 30°, 45°, and 60° (Figure S7). Among them, the LSPR peak obtained from Au (45°)/PSS showed the sharpest. This work is a preliminary study demonstrating the application of PSS for LSPR substrate by obliquely depositing Au on the PSS, and thus, LSPR properties of the Au nanostructure on PSS can be improved further, depending on Au deposition angle, the thickness of deposited Au, the direction of PSS under oblique deposition of Au, and the shape of PSS. 

## 4. Conclusions

In this paper, we have suggested a fabrication method of wafer-scale LSPR substrates by engineering a single crystal substrate and obliquely depositing Au, using high-throughput and high-reproducibility techniques. As a single crystal substrate, sapphire wafers were chosen because of their transparency, which is highly advantageous for optical applications, such as optical biosensors, metamaterials, and solar cells. The PSS was fabricated using a nanoimprint lithography technique, thin film deposition, and wet-etching (using H_2_SO_4_ and H_3_PO_4_). The oblique deposition (45°) of Au on the PSS enabled the formation of Au nanostructures on the PSS due to the three-dimensional structure (triangular-pyramid shape) of the PSS. The Au nanostructures formed by the oblique deposition exhibited a red-shifted LSPR peak compared to those prepared by vertical deposition, implying that LSPR properties can be controlled not only by the shapes of the PSS but also by the deposition conditions, such as angle and/or rotation of the PSS. The Au nanostructure on PSS was applied to detect biomolecules by observing the LSPR shift; the LSPR peak was red-shifted by attaching STA, biotin-BSA, and STA, in turn. 

PSS has been widely used as a substrate for light-emitting diode, so far due to its transparency, wafer-scale patterning, and high-throughput production. Here, we applied such a PSS to the fabrication of an LSPR substrate by obliquely depositing Au on the PSS. Further technical approaches could improve our fabrication strategy to expedite its application for commercially available products. As an example of the approaches, some experimental variables—Au deposition angle, the thickness of deposited Au, the direction of PSS under oblique deposition of Au, and the shape of PSS—could be addressed. In addition, due to the high expense of the sapphire wafer, the PSS might be used as a master mold to fabricate the reverse-shaped polydimethylsiloxane (PDMS) that could be used as patterned PDMS substrate for the angle deposition of Au.

Thus, such a fabrication method of wafer-scale plasmonic substrates could pave the way for use in LSPR biosensors by manipulating the structures of the PSS and/or oblique deposition conditions. This technology is also expected to be useful in other optical applications, such as color engineering, spectroscopy, metamaterials, and plasmonic optoelectronics.

## Data Availability

The authors confirm that the data supporting the findings of this study are available within the article and its Supplementary Materials.

## Supplementary Materials

The following supporting information can be downloaded at: https://www.mdpi.com/article/10.3390/bios12030158/s1, Figure S1: Schematic of the formation of a SiO_2_ nanodot array on a sapphire wafer, Figure S2: SEM image of the SiO_2_ nanodot (not annealed)-patterned sapphire wafer after the wet-etch, Figure S3: SEM images of a PSS, Figure S4: SEM images after the second wet-etch of PSS, Figure S5: SEM images of vertically deposited Au on PSS. Figure S6: Schematic illustration of vertical and oblique depositions of Au on PSS, Figure S7: Analysis of Au/PSSs where Au was deposited at different angles, Table S1: LSPR-shift observation of obliquely deposited Au on PSS when exposed to biomolecules.
