# Supplementary material for: Wafer-Scale LSPR Substrate: Oblique Deposition of Gold on a Patterned Sapphire Substrate
Source: Biosensors (Basel). 2022 Mar 3;12(3):158. doi: 10.3390/bios12030158 (PMC8946711; doi:10.3390/bios12030158)
Supplement: Supplementary file 1 [file biosensors-12-00158-s001.zip › biosensors-1623155-supplementary.pdf]

biosensors

Supplementary Materials

Wafer-Scale LSPR Substrate: Oblique Deposition of Gold on a Patterned Sapphire Substrate

Kihyeun Kim [1,†], Ki Joong Lee [2,†], Na Rae Jo [2], Eun-Jung Jo [3], Yong-Beom Shin [2,4,*] and Min-Gon Kim [3,*]

[1] Center for Systems Biology, Massachusetts General Hospital, Boston, MA 02114, USA; kkim50@mgh.harvard.edu
[2] Bionanotechnology Research Center, Korea Research Institute of Bioscience and Biotechnology (KRIBB), 125 Gwahak-ro, Yuseong-gu, Daejeon 34141, Korea; kijoong123@kribb.re.kr (K.J.L.); nalgamgi@kimm.re.kr (N.R.J.)
[3] Department of Chemistry, Gwangju Institute of Science and Technology (GIST), Gwangju 61005, Korea; jej@gist.ac.kr
[4] BioNano Health Guard Research Center, 125 Gwahak-ro, Yuseong-gu, Daejeon 34141, Korea
* Correspondence: ybshin@kribb.re.kr (Y.-B.S.); mkim@gist.ac.kr (M.-G.K.)
† These authors contributed equally to this work.

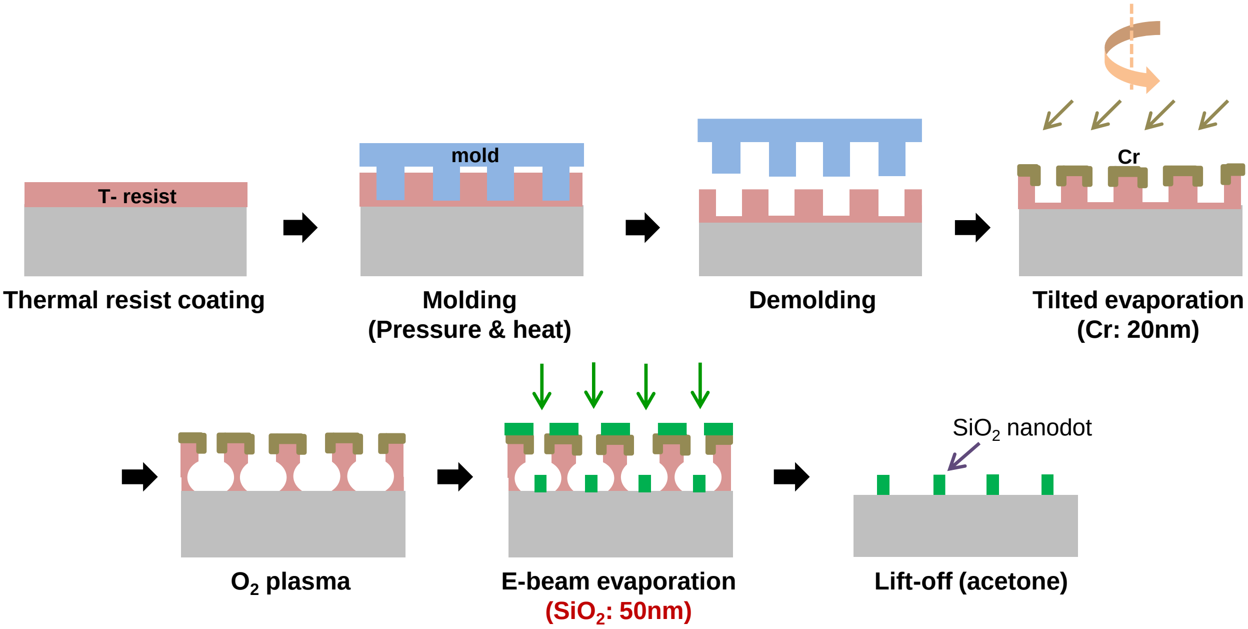

Figure S1. Schematic of the formation of a SiO_2 nanodot array on a sapphire wafer using nanoimprint lithography.

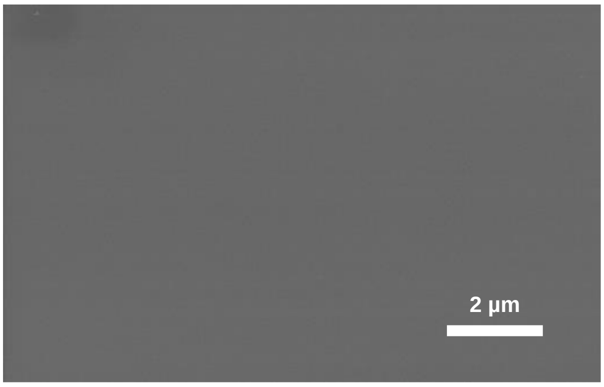

Figure S2. SEM image of the SiO_2 nanodot (not annealed)-patterned sapphire wafer after the wet etch. This image indicates that SiO_2 nanodots were etched by H_2SO_4 and H_3PO_4, which was not able to act as hard masks.

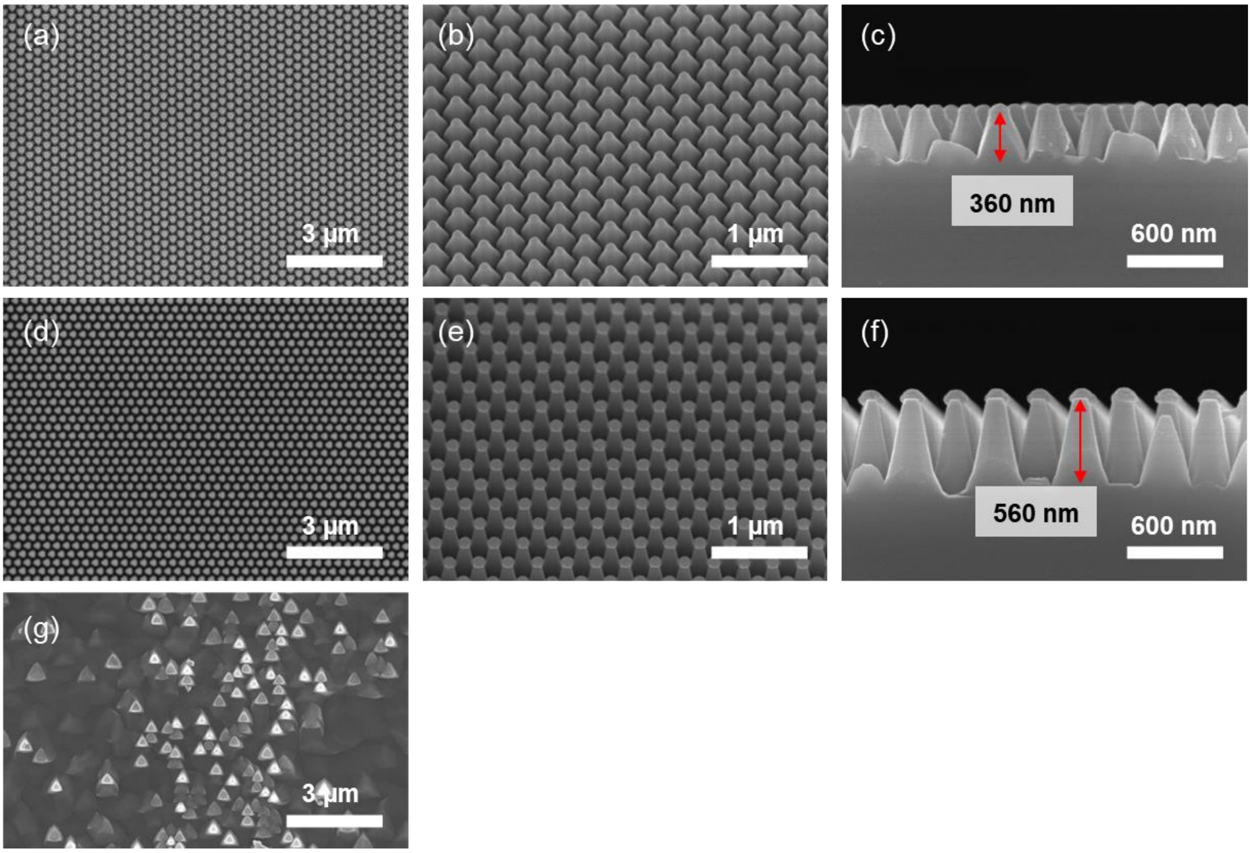

Figure S3. SEM images of a PSS (wet etch solution: H_2SO_4:H_3PO_4 = 3:1 (v/v)): etch temperature: (a-c) 310°C, (d-f) 330°C, and (g) 350°C. (a),(d),(g) Top, (b),(e) 45° tilted, and (c),(f) side views.

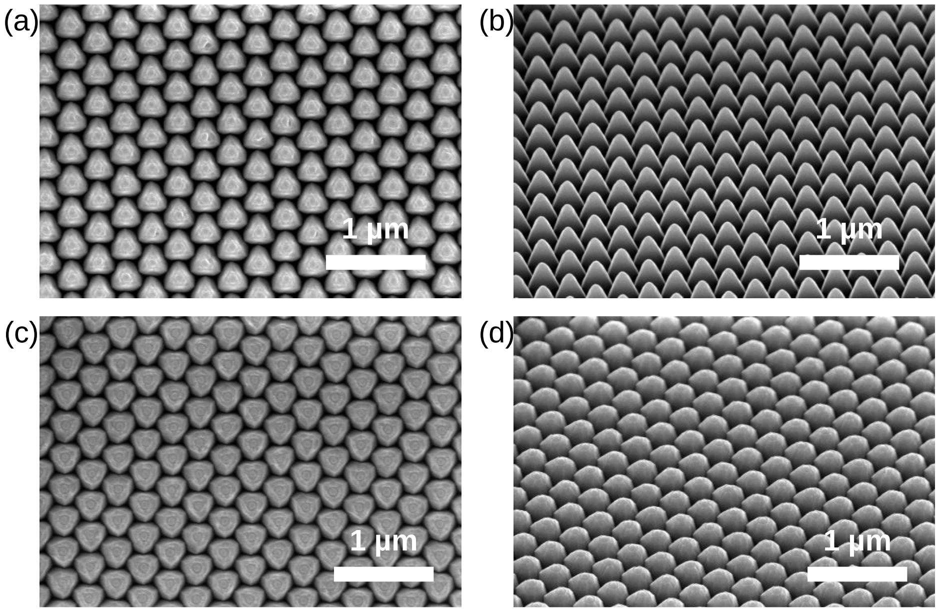

Figure S4. SEM images after the second wet etch of PSS. Before the second wet etch, SiO_2 hard masks were eliminated using a buffered oxide etch solution. Conditions for the second wet etches were (a), (b) H_2SO_4:H_3PO_4 = 1:1 and (c), (d) H_2SO_4:H_3PO_4 = 1:3, at 310°C and 40 min for both. ((a) and (c): top views, (b) and (d): 45° tilted views).

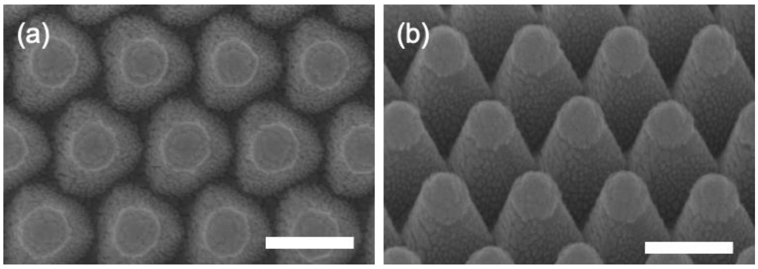

Figure S5. SEM images after vertical deposition of Au onto a PSS ((a): top view, (b): 45° tilted view). Scale bars in the images are 300 nm.

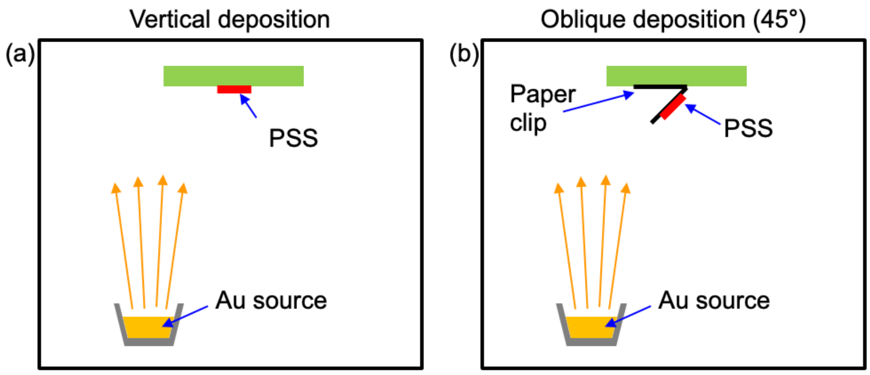

Figure S6. Schematic illustration of (a) vertical deposition and (b) oblique deposition of Au on PSS.

Figure S7. Absorbance and SEM images of Au/PSS where Au was deposited at the angle of (a) 0°, (b) 30°, (c) 45°, and (d) 60° (Note that (i), (ii), and (iii) indicate absorbance data, top-view SEM images, and tilted-view, 45°, SEM images, respectively.).

Table S1. LSPR-shift observation of obliquely deposited Au on PSS when exposed to biomolecules.

**Adding sequence**	**1**	**2**	**3**	**4**
Added molecule	Biotin-HPDP	STA	Biotin-BSA	STA

LSPR peak (nm)	782	784	786	788
Shift (Δ)	-	+2	+2	+2
